# Bronchitis, and Kindred Diseases

**Published:** 1854-05

**Authors:** 


### THE AIR PASSAGES.

The following sixteen pages are a verbatim monograph of the edition published several years ago, treating of some of the diseases which affect the throat and lungs. By using small type the whole is presented in one number of the *Journal*, otherwise it would require several entire numbers:—

### BRONCHITIS, AND KINDRED DISEASES.

BY

W. W. HALL, A. M., M. D., NEW YORK.

There is no necessary reason why men should not generally live to the full age of three score years and ten, in health and comfort: that they do not do so, is because

THEY CONSUME TOO MUCH FOOD, AND TOO LITTLE PURE AIR;
THEY TAKE TOO MUCH MEDICINE, AND TOO LITTLE EXERCISE:

and when, by inattention to these things, they become diseased, they die chiefly, not because such disease is necessarily fatal, but because the symptoms which nature designs to admonish of its presence, are disregarded, until too late for remedy. And in no class of ailments are delays so uniformly attended with fatal results, as in affections of the Throat and Lungs. However terrible may have been the ravages of the Asiatic Cholera in this country, I know of no locality, where, in the course of a single year, it destroyed ten per cent. of the population. Yet, taking England and the United States together, twenty per cent. of the mortality is every year from diseases of the lungs alone; amid such a fearful fatality, no one dares say he shall certainly escape, while every one, without exception, will most assuredly suffer, either in his own person, or in that of some one near and dear to him, by this same universal scourge. No man, then, can take up these pages, who is not interested to the extent of life and death, in the important inquiry, *What can be done to mitigate this great evil?* It is not the object of this publication to answer that question; but to act it out; and the first great essential step thereto, is to impress upon the common mind, in language adapted to common readers, a proper understanding of the first symptoms of these ruthless diseases

Every reader of common intelligence and of the most ordinary observation, must know that countless numbers of people in every direction have been saved from certain death by having understood the premonitory symptoms of Cholera, and acting up to their knowledge. The physician does not live, who, in the course of ordinary practice, cannot point to a little army of the prematurely dead who have paid the forfeit of their lives by ignorance or neglect of the early symptoms of Consumptive disease. Perhaps the reader's own heart is this instant smitten at the sad recollection of similar cases in his own sphere of observation.

This book is not intended to recommend a medicinal preventive, or a patented cure for the diseases named on the title-page: it will afford no aid or comfort to those who hope, by its perusal, to save a doctor's fee, by a trifling tampering with their constitutions and their lives. Nor is it wished to make you believe, that if you come to me I will cure you. If you have symptoms of disease, I wish you to understand their nature first; and then to take advice from some regularly educated physician, who has done nothing to forfeit justly his honorable standing among his brethren, by the recommendation of secret medicines, patented contrivances or travelling lecturers for the cure of certain diseases. I may speak of persons in these pages, who had certain symptoms, and coming to me, were permanently cured. You may have similar symptoms, and yet I may be able to do you no good. I have sometimes failed to cure persons who had no symptoms at all. In other cases, where but a single symptom of disease existed, and it, apparently, a very trivial one, the malady has steadily progressed to a fatal termination, in spite of every effort to the contrary. The object of these statements is to have it understood, that I make no engagement to cure any thing or any body. The first great purpose is to enable you to understand properly any symptoms which you may have that point towards disease of the lungs; and when you have done so, to persuade you not to waste your time and money and health in blind efforts to remove them, by taking stuff, of which you know little, into a body of which you know less; but to go to a man of respectability and standing and experience—one in whom you have confidence, one who depends upon the practice of his profession for a living; describe your symptoms, according to your ability, place your health and life in his hands, and be assured that thus you and millions of others will stand the highest chance of attaining a prosperous, cheerful, and green old age. The rule should be universal, and among all classes, not only never to take an atom of medicine for anything, but not *to take anything as a* MEDICINE—not even a teaspoon of common syrup or French brandy, or a cup of red pepper tea, unless by the previous advice of a physician; because a spoonful of the purest, simplest syrup, taken several times a day, will eventually destroy the tone of the healthiest stomach: and yet any person almost would suppose that a little syrup "*could do no harm, if it did no good.*" A tablespoon of good brandy, now and then, is simple enough, and yet it has made a wreck and ruin of the health and happiness and hope of multitudes. If these *simple*, that is, *well-known things, in their purity*, are used to such results, it requires but little intelligence to understand that more speedy injuries must follow their daily employment, morning, noon, and night, when they are sold in the shape of "syrups," and "bitters," and "tonics," with other ingredients, however "*simple*" they, too, may be.

The common-sense reader will consider these sentiments reasonable and right, and think it a very laudable desire to diffuse information among the people as to the symptoms of dangerous, insidious, and wide-spreading diseases; but he will not be prepared for the information, that the publication of such a pamphlet as this will be considered "unprofessional" by some. But latitude must be allowed for difference of opinion; else, all progress is at an end. Whoever lends a helping hand to the diffusion of useful knowledge, is, in proportion, the benefactor of his kind. Whether it be useful for man to know the nature and first symptoms of a disease which is destined to destroy one out of every six in the country, is a question which each one must decide for himself. I believe that such an effort is useful, and hereby act accordingly. Experienced physicians constantly feel, in reference to persons who evidently have Consumption, that it is too late, because the application had been too long delayed. The great reason why so many delay, is because they "did not think it was anything more than a slight cold" In other words, they were entirely ignorant of the difference between the cough of a common cold and the cough of Consumption, and the general symptoms attendant on the two. It is not practicable for all to study medicine, nor is it to be expected that for every cough one has, he shall go to the expense of taking medical advice; it therefore seems to me the dictate of humanity to make the necessary information more accessible, and I know of no better way to accomplish this object than by the general distribution of a tract like this: and when I pretend to no new principle of cure, no specific, and no ability of success, beyond what an entire devotion to one disease may give any ordinary capacity, no further apology is necessary.

### THROAT-AIL, or Laryngitis, pronounced *Lare-in-*GEE*-tis*, is an affection of the top of the windpipe, where the voice-making organs are, answering to the parts familiarly called "Adam's Apple." When these organs are diseased, the voice is impaired, or "*there is something wrong about the swallow.*"

### BRONCHITIS, pronounced *Bron-*KEE*-tis*, is an affection of the *branches* of the windpipe, and in its first stages is called a common cold.

### CONSUMPTION

is an affection, not of the *top* or *root* of the windpipe, for that is *Throat-Ail;* not of the *body* of the windpipe, for that is *Croup;* not of the *branches* of the windpipe, for that is *Bronchitis;* but it is an affection of the lungs themselves, which are millions of little air cells or bladders, of various sizes, from that of a pea downwards, and are at the *extremities* of the branches of the windpipe, as the buds or leaves of a tree are at the extremity of its branches.

#### WHAT ARE THE SYMPTOMS OF THROAT-AIL?

The most universal symptom is an impairment of the voice, which is more or less hoarse or weak. If there is no actual want of clearness of the sounds, there is an instinctive clearing of the throat, by swallowing, hawking, or hemming; or a summoning up of strength to enunciate words. When this is continued for some time, there is a sensation of tiredness about the throat, a dull heavy aching, or general feeling of discomfort or uneasiness, coming on in the afternoon or evening. In the early part of the day, there is nothing of the kind perceptible, as the voice-muscles have had time for rest and the recovery of their powers during the night. In the beginning of this disease, no inconvenience of this kind is felt, except some unusual effort has been made, such as speaking or singing in public; but as it progresses, these symptoms manifest themselves every evening; then earlier and earlier in the day, until the voice is clear only for a short time soon in the morning; next, there is a constant hoarseness or huskiness from week to month, when the case is most generally incurable, and the patient dies of the common symptoms of Consumptive disease.

In some cases, the patient expresses himself as having a sensation as if a piece of wool or blanket were in the throat, or an aching or sore feeling, running up the sides of the neck towards the ears. Some have a burning or raw sensation at the little hollow at the bottom of the neck; others, about Adam's Apple; while a third class speak of such a feeling or a pricking at a spot along the sides of the neck. Among others, the first symptoms are a dryness in the throat after speaking or singing, or while in a crowded room, or when waking up in the morning. Some feel as if there were some unusual thickness or a lumpy sensation in the throat, at the upper part, removed at once by swallowing it away; but soon it comes back again, giving precisely the feelings which some persons have after swallowing a pill.

Sometimes, this frequent swallowing is most troublesome after meals. Throat-Ail is not like many other diseases, often getting well of itself by being let alone. I do not believe that one case in ten ever does so, but on the contrary, gradually grows worse, until the voice is permanently husky or subdued; and soon the swallowing of solids or fluids becomes painful, food or drink returns through the nose, causing a feeling of strangulation or great pain. When Throat-Ail symptoms have been allowed to progress to this stage, death is almost inevitable in a very few weeks. Now and then a case may be saved, but restoration here is almost in the nature of a miracle.

### WHAT ARE THE SYMPTOMS OF BRONCHITIS?

Bronchitis is a bad cold, and the experience of every one teaches what its symptoms are. The medical name for a cold is *Acute Bronchitis;* called *acute*, because it comes on at once, and lasts but a short time—a week or two generally. The ailment that is commonly denominated *Bronchitis*, is what physicians term *Chronic Bronchitis;* called *chronic*, because it is a long time in coming on, and lasts for months and years instead of days and weeks. It is not like Throat-Ail, or Consumption, which have a great many symptoms, almost any one of which may be absent, and still the case be one of Throat-Ail, or Consumption; but Bronchitis has three symptoms, every one of which are present every day, and together, and all the time, in all ages, sexes, constitutions, and temperaments. These three universal and essential symptoms are—

1st. A feeling of fullness, or binding, or cord-like sensation about the breast.

2d. A most harassing cough, liable to come on at any hour of the day or night.

3d. A large expectoration of a tough, stringy, tenacious, sticky, pearly or greyish-like substance, from a tablespoon to a pint or more a day. As the disease progresses, this becomes darkish, greenish, or yellowish in appearance; sometimes all three colors may be seen together, until at last it is uniformly yellow, and comes up without much effort, in mouthfuls, that fall heavily, without saliva or mucus. When this is the case, death comes in a very few weeks or—days.

### WHAT ARE THE SYMPTOMS OF CONSUMPTION?

A gradual wasting of breath, flesh, and strength are the three symptoms, progressing steadily through days and weeks and months, which are never absent in any case of true, active, confirmed Consumptive disease that I have ever seen. A man may have a daily cough for fifty years, and not have Consumption. A woman may spit blood for a quarter of a century, and not have Consumption. A young lady may breathe forty times a minute, and have a pulse of a hundred and forty beats a minute, day after day, for weeks and months together, and not have Consumption; and men and women and young ladies may have pains in the breast, and sides, and shoulders, and flushes in the cheeks, and night sweats, and swollen ankles, and yet have not an atom of Consumptive decay in the lungs. But where there is a slow, steady, painless decline of flesh and strength and breath, extending through weeks and months of time, Consumption exists in all persons, ages, and climes, although at the same time sleep, bowels, appetite, spirits, may be represented as good. Such, at least, are the results of my own observation.

The great, general, common symptoms of Consumption of the Lungs are night and morning cough, pains about the breast, easily tired in walking, except on level ground, shortness of breath on slight exercise, and general weakness. These are the symptoms of which Consumptive persons complain, and as they approach the grave, these symptoms gradually increase.

### HOW DOES A PERSON GET THROAT-AIL?

A woman walked in the Park, in early spring, until a little heated and tired; then sat down on a cold stone. Next day, she had hoarseness and a raw burning feeling in the throat, and died within the year.

A man had suffered a great deal from sick headache; he was advised to have cold water poured on the top of his head: he did so; he had headache no more. The throat became affected; had frequent swallowing, clearing of throat, falling of palate, voice soon failed in singing, large red splotches on the back part of the throat, and white lumps at either side; but the falling of the palate and interminable swallowing were the great symptoms, making and keeping him nervous, irritable, debilitated, and wretched. He was advised to take off the uvula, but would not do it. Had the nitrate of silver applied constantly for three months. Tried homœopathy. After suffering thus two years, he came to me, and on a subsequent visit, said, "It is wonderful, that for two years I have been troubled with this throat, and nothing would relieve it, and now it is removed in two days." That was four months ago. I saw him in the street yesterday. He said his throat gave him no more trouble; that he had no more chilliness, and had never taken a cold since he came under my care, although formerly "it was the easiest thing in the world to take cold."

A merchant (1002) slept in a steamboat state-room in December, with a glass broken out; woke up next morning with a hoarseness and sore throat; for several months did nothing, then applied to a physician. Counter-irritants were employed without any permanent effect. At the end of four years, he came to me with "a sort of uneasy feeling about the throat, more at times than others; not painful; sometimes a little hoarseness, with frequent inclination to swallow, or clear the throat. At the little hollow at the bottom of the neck, just above the top of the breast-bone, there was a feeling of pressure, stricture, or enlargement—no pain, but an unpleasant sensation, sometimes worse than at others. It is absent for days at a time, and then lasts for several hours a day." This case is under treatment.

A Clergyman (1012) has a hoarse, cracked, weak voice, easily tired in speaking; a raw sensation in the throat; and in swallowing has "*a fish-bony feeling*." He had become over-heated in a public address, and immediately after its close started to ride across a prairie in a damp, cold wind in February. Had to abandon preaching altogether, and become a school teacher." This gentleman wrote to me for advice, and having followed it closely for eighteen days, reported himself as almost entirely well.

I greatly desire it to be remembered here, that in this, as in other cases of Throat-Ail, however perfectly a person may be cured, the disease will return as often as exposure to the causes of it in the first place is permitted to occur. No cure, however perfect, will allow a man to commit with impunity such a thoughtless and inexcusable act as above named, that of riding across a prairie in February, in a damp, cold wind, within a few minutes after having delivered an excited address in a warm room. None of us are made out of India rubber or iron, but of flesh and blood and a reasonable soul, subject to wise and benevolent conditions and restrictions; and it is not to the discredit of physic or physicians, that being once cured, the disease should return as often as the indiscretion that originated it in the first instance is re-committed.

Three weeks ago, one of our merchants came to me with a troublesome tickling in the throat. At first it was only a tickling; but for some weeks the tickling compels a frequent clearing of the throat; and without a cough, each clearing or hemming brings up half a teaspoon-ful of yellow matter, with some saliva. On looking into his throat, the whole back part of it was red, with still redder splotches here and there—epiglottis almost scarlet. On inquiry, I found he had for years been a chewer of tobacco; then began to smoke; would day after day smoke after each meal, but especially after tea would consume half a dozen cigars. In time, the other naturally consequent steps would have been taken—Consumption and the grave. Among other things, I advised him to abandon tobacco absolutely and at once. In two weeks he came again. Throat decidedly better; in every respect better, except that he, in his own opinion, "had taken a little cold," and had a constant slight cough—not by any means a trifling symptom. Let the reader learn a valuable lesson from this case. This gentleman had the causes of cough before; he found that smoking modified the tickling, and taking this as an indication of cure, he smoked more vigorously, and thus suppressed the cough, while the cause of it was still burrowing in the system and widening its ravages. It will require months of steady effort to arrest the progress of the disease, and he may consider himself fortunate—more so than in any mercantile speculation he ever made—if he gets well at all. If he does get well, and returns to the use of tobacco, the disease will as certainly return as that the same cause originated it, for the following reason, as was stated in the *First Part:*—Throat-Ail is inflammation; that is, too much heat in the parts. Tobacco smoke being warm, or even hot, is drawn directly back against the parts already too much heated, and very naturally increasing the heat, aggravates the disease. Again, any kind of smoke—that of common wood—is irritating, much more that of such a powerful poison as tobacco

—soothing, indeed, in its first transient effects, like many other poisons, but leaving behind it consequences more remote, but more destructive and enduring.

A gentleman, just married, with a salary for his services as secretary to a Southern house, applied to me to be cured of a sore throat. He was permanently hoarse; swallowing food was often unendurably painful, besides causing violent paroxysms of cough. He said he knew no cause for his complaint, except that he had smoked very freely. On inquiry, I found that for the last two years he had used, on an average, about "a dozen cigars every day; perhaps more." He died in six weeks.

In several instances, persons have applied to me who had been advised to take brandy freely for a throat affection. Such advice is warranted by no one principle in medicine, reason, or common sense. Were I to give it, I should feel myself justly liable to the charge of being an ignorant man or a drunkard. The throat is inflamed; inflammation is excitement; brandy and tobacco both excite, inflame the whole body; that is why they are used at all. The throat partakes of its portion of the excitement, when the throat, body, and the man, all the more speedily go to ruin together. I have in my mind, while writing these lines, the melancholy history of two young men—one from Kentucky, the other from Missouri—who were advised "to drink brandy freely, three times a day, for throat complaint." One of these became a drunkard, and lost his property, and within another year he will leave an interesting family in penury, disgrace, and want. The other was one of the most high-minded, honorable young men I have lately known. He was the only son of a widow, and she was rich. He came to see me three or four times, and then stated that he had concluded to try the effects of a little brandy at each meal. A few weeks afterwards he informed me, that as he was constantly improving, he thought that the brandy would certainly effect a cure. Within seven months after his application to me, he had become a regular toper; that is, he had increased the original quantity allowed, of a tablespoon at each meal, to such an amount, that he was all the time under the influence of liquor. His business declined; he spent all his money; and secretly left for California, many thousand dollars in debt, and soon after died. The person who advised him is also now a confirmed drunkard; but in his wreck and ruin, still a great man.

A gentleman from a distant State wrote to me some months ago for advice as to a throat affection. He is a lawyer of note already, and of still higher promise, not yet having reached the prime of life. By earnest efforts as a temperance advocate, in addition to being a popular pleader at the bar, his voice became impaired with cough, spitting of blood, matter expectoration, diarrhœa, debility, and general wasting. He was induced to drink brandy with iron, but soon left off the iron and took the brandy pure. The habit grew upon him; he *sometimes* stimulated to excess, according to his own acknowledgment; his friends thought there was no interval, and gave him up as a lost man to themselves, his family, and his country; but in time the virulence of the disease rose above the stimulus of the brandy, and in occasional desperation he resorted to opium. He subsequently visited the water cure, gained in flesh and strength, and was hopeful of a speedy restoration; but he took "an occasional cigar"—the dryness in the throat, hoarseness, pain or pressure, and soreness still remained! He left the water cure, and in a few months wrote to me, having, in addition to the above throat symptoms, a recent hæmorrhage, constipation, pains in the breast, nervousness, debility, variable appetite, and daily cough. Within two months, he has become an almost entirely new man, requiring no further advice.

Further illustrations of the manner in which persons get Throat-Ail, may be more conveniently given in the letters of some who have applied to me, with the additional advantage of having the symptoms described in language not professional, consequently more generally understood.

### A PRESBYTERIAN CLERGYMAN.

(1059.) "I have had for three years past a troublesome affection of the thorax, which manifests itself by frequent and prolonged hemming or clearing the throat, and swelling: both more frequent in damp weather, or after slight cold. General health very feeble, sleeplessness, waste of flesh, low spirits. Visited a water cure, remained two months, but my hemming and swallowing were not a whit improved. Touching with the nitrate of silver slightly makes the larynx sore. I have been always able to preach. It has never affected my voice until very recently. Two weeks ago I preached two long sermons, in a loud and excited voice, in one day. During the last discourse my voice became hoarse, and my hemming has become very bad; and there has been a slight break in my voice ever since. Hem, hem, hem, is the order of the day; clearing the throat is incessant, swallowing often, and a slight soreness of the larynx, particularly after a slight cold, or after several days' use of nitrate of silver, with a scarce perceptible break in the voice. These are my principal symptoms."

This case is under treatment.

### A LAWYER,

(1016) "aged thirty-seven. Have been liable, for several years past, in the fall, winter, and spring, to severe attacks of fever, accompanied with great debility, loss of flesh, appearing to myself and friends to be in the last stages of Consumption; in fact, the dread of it has been an incubus on me, paralyzing my energies and weighing down my spirits. In the summers, too, I have been subject to attacks of bilious fever and bilious colic. A year ago, I attended court soon after one of these attacks, and exerted myself a great deal. My throat became very sore, and I had hemorrhage—two teaspoons of blood and matter. My health continued feeble. I went last summer to a water cure, and regained my flesh and strength, but the weakness in my throat and occasional hoarseness continued all the time. Afterwards, by cold and exposure, I became worse, continued to have chills and fever and night sweats, accompanied by violent cough and soreness of the throat. I got worse; was reduced to a perfect skeleton, and had another hæmorrhage. Mucus would collect in the top of the throat, and was expectorated freely. I am still liable to colds. The seat of the disease seems to be at the little hollow in front at the bottom of the neck, just above the top of the breast-bone. At my last bleeding, the pain seemed to be in the region of Adam's-apple. The principal present symptoms are soreness in throat, dryness, pain on pressing it, and hoarseness; pulse from eighty to ninety in a minute; irregular appetite. These symptoms, together with my fear of Consumption, serve to keep me unhappy. I find myself constantly liable to attacks of cold, sneezing, running at the nose even in the summer time. My mother and sister have died of Consumption, as also two of my mother's sisters. Feet always cold; daily cough."

### OPINION OF THE CASE.

There is no Consumptive disease; it is impossible. No personal examination is needed to tell that. The foundation of all your ailments is a torpid liver and a weak stomach. If you are not cured, it will be your own fault.

The treatment of this case was conducted by correspondence, as he lived six hundred miles away, and therefore I had not the opportunity of a personal examination. Within a month he writes:—"I am gradually improving; feet warm; all pain has disappeared from the breast; appetite strong, regular, and good: pulse seventy-two; breathing eighteen; all cough has disappeared." At the end of two and a half months, no further advice was needed, as he wrote—"I have not written to you for a month, being absent on the circuit. I have not enjoyed better health for years than I have for the month. Weight increasing; no uneasiness or pain about my breast; pulse seventy-five; less in the morning. The only trouble I have is costiveness, from being so confined in court, and being away from home deprived of my regular diet. We were two weeks holding court, last of November, in a miserable room, the court-house having been recently burned; kept over-heated all the time. I made four or five speeches, and suffered no inconvenience whatever. I have no cough."

### A CLERGYMAN

(1024) called over two months ago, having had at first an ailment at the top of the throat, apparently above or near the palate. It soon descended to the region of Adam's-apple, and within a month it seemed to have located itself lower down the neck, giving a feeling as if there were an ulcer there, with a sense of fullness about the throat, hoarse after public speaking, lasting a day or two, with attacks every few weeks of distressing sick headache. As the disease seemed to be rapidly descending towards the lungs, a rigid, energetic treatment was proposed, and at the end of ten weeks he writes—"I take pleasure in introducing my friend, ——, to you. He has suffered many things, from many advisers, with small benefit. I have desired him to consult with you, hoping that he may have the same occasion to be grateful for the providence which leads him to you, which I feel that I myself have for that which guided me to your counsels. I suffer but little, very little from my throat, and confidently anticipate entire relief at no distant day, for all which I feel myself under great obligation both to your skill and to your kindness," &c.

### SICK HEADACHE

is a distressing malady, as those who are subject to it know full well, by sad experience. In this case, this troublesome affection had to be permanently removed before the throat ailment could be properly treated; when that was done, the throat itself was comparatively of easy management.

### A MERCHANT

(947) wrote to me from the South, complaining chiefly of

Bad cough, sometimes giving a croupy sound;
Throat has a raw, choking, dry, rasping feeling;
Soon as he goes to sleep, there is a noise or motion, as if he were going to cough;
Startled in sleep, by mouth filling with phlegm;
Expectoration tough, white, and sticky; darkish particles sometimes;
Flashes or flushes pass over him sometimes;
Sick stomach sometimes, acid often, wind on stomach oppresses him greatly;
A lumpy feeling in the throat;
On entering his house, sometimes falls asleep in his chair, almost instantly;
In walking home, at sundown, half a mile from his store, is completely exhausted;
Slightest thing brings on a cough; never eats without coughing;
If he swallows honey, it stings the throat;
Got a cold a month ago, which left the palate and throat very much inflamed;
Throat and tongue both sore;
A hooping, suffocative cough; can hear the phlegm rattle just before the cough begins;
A dry, rough feeling from the little hollow at the bottom of the neck up to the top of the throat.
One night after going to bed, began to cough, choke, suffocate; could not get breath, jumped out of bed, ran accross the room, struggled, and at length got breath, but was perfectly exhausted; could not speak for half an hour, without great difficulty.

In addition to his own description of the case, his wife writes—"Ten o'clock at Night.—I am no physician, nor physician's wife, but am his wife and nurse, and an anxious observer of his symptoms, and can see his throat inflamed behind the uvula. He says there is a lump somewhere, but he cannot tell where. Sometimes he thinks it is in the little hollow at the bottom of the neck, sometimes just above, and sometimes in or about the swallow. A recent cold has aggravated his symptoms. His cough to-day has been very frequent and loose. He has emaciated rapidly within a month, and is now a good deal despondent. As for myself, I feel as one who sees some fair prospect suddenly fading away. I had fondly hoped—oh! how ardently!—that he might be restored. If a knowledge of the fact would give any additional interest to the case, I will only say, he is one of the loveliest characters on earth. None in this community has a larger share of the respect and confidence of their acquaintance."

The opinion sent, for I have not seen this case, was as follows:—"The whole breathing apparatus, from the top of the windpipe to the extremity of its branches, is diseased; the lungs themselves are not at all affected by decay. Your whole constitution is diseased; and yet there is good ground for hope of life and reasonable health."

In three months this patient writes—"I am glad to inform you that I think I am still improving in health and strength. My bowels are sometimes disordered by eating melons and fruits; but I felt so much better that I thought I might indulge. Pulse sixty-five to seventy; an almost ravenous appetite." A month later he writes—"My health and strength are still improving; cough not very troublesome; increasing in flesh," &c. I believe this gentleman now enjoys good health.

### A LADY.

(948) teacher of vocal music, writes—"There is a peculiar sensation in my throat for the last two months. Whenever I attempt to swallow, it feels as if something were in the way; a swelling under the jaws, a soreness on the sides of the throat, extending to the ears, and occasioning throbbing painfully. I have a dull aching at the top of my collar-bone, and an unpleasant sensation of weakness and heaviness in my chest; a bad taste in my mouth frequently. Have been regular, but have been afflicted for a few years past with sickness at the stomach and vomiting, attended occasionally with great pain for a few hours. During these attacks, the complexion changes to a livid hue. I have been very much troubled with dyspepsia. On recovering from the attacks above mentioned, I have experienced a feeling of weakness almost insupportable. Am very costive; and my spirits are greatly depressed. Within a day or two I have taken a violent cold, which has affected me with sneezing, running from the eyes and nose, together with a slight hoarseness. I was advised to apply caustic to the throat, and Croton oil to my neck, chest, and throat. I have since discontinued these, not having received any permanent benefit from them. On two occasions, from over-exertion at concerts and examinations, I was unable to speak a loud word, from hoarseness, for several days. I am extremely anxious to learn your opinion. In about two months my public concerts take place, and it is absolutely necessary that something should be done for me."

### OPINION.

Yours is general constitutional disease. There is no special cause of alarm. A weakened stomach, a torpid liver, a want of sufficient air and exercise, are the foundations of all your ailments, and by the proper regulation of these, you may expect to have good health and a stronger voice. You must have energy and patient perseverance in carrying out the prescriptions sent to you.

In one month this lady writes, and the letter is given to encourage others who may come under my care, to engage with determination and energy in carrying out the directions which may be given them. The reader may also see what great good a little medicine may do when combined with the judicious employment of rational means, which do not involve the taking of medicine or the use of painful and scarifying agencies and patent contrivances:—

"I began your prescriptions at once. Having followed them for some time, I was obliged to intermit them for a few days, in consequence of having to conduct a concert, besides having to travel by stage and railroad seventy or eighty miles. During this time, I was up every night until twelve o'clock, and was much exposed to the night air. On returning home, I re-commenced your directions, have made it a point to attend to them strictly, and have very seldom failed of doing so. In consequence of two omissions in diet, I suffered from headache, which disappeared when I observed your directions. My appetite is good; my food agrees with me. I sometimes feel dull and sleepy after dinner. I drop to sleep immediately. Seldom wake in the night. Sleep about seven hours, and generally feel bright and strong in the morning, when I take a brisk walk of two miles and a half; the same after six, P.M. My walks at first fatigued me considerably; generally, however, I have felt better and better from their commencement to their end, and have perspired very freely. The exercise I take seems rather to increase than diminish my strength. I have not been prevented from taking exercise from any dampness in the atmosphere. I have sometimes been exposed to the night air in going to church and other places, but without any perceptible injury. The means you advised produce a general glow, and invariably remove headache, which I sometimes have to a slight degree after dinner. I think my throat is better. There is no unpleasant feeling about it at present, except the difficulty in swallowing, and even that is better. Pulse sixty-seven."

I had for some time ceased to regard this energetic young lady as a patient, when she announces a new ailment, a difficulty at periodic times:—"I walked two miles every day, and every thing was going on well, until one evening after walking very fast, I sat awhile with a friend, in a room without fire, in November. The weather was chilly and damp; was unwell, suppressed; had a chill and incessant cough for several hours, ending in something like inflammation of the lungs."

These things were remedied, and she is now engaged in the active discharge of her duties. This last incident is introduced here to warn every reader, especially women, against all such exposures at all times, most especially during particular seasons. Such exposures, as sitting in rooms without fire, in the fall and spring, after active walking, have thrown stout strong men into a fatal consumption; and it is not at all to be wondered at that delicate women should lay the foundation of incurable disease in the same manner. I will feel well repaid for writing these lines, if but here and there a reader may be found to guard against such exposures. Our parlors and drawing-rooms are kept closed to the air and light for a great portion of the twenty-four hours, and unless the weather is quite cool there is no fire in them. Thus they necessarily acquire a cold, clammy dampness, very perceptible on first entering. A fire is not thought necessary, as visitors usually remain but a few minutes; but when the blood is warmed by walking in the pure air and the clear sunshine, it is chilled in a very short space of time, if the person is at rest, in the cold and gloom of a modern parlor, especially as a contemplated call of a minute is often unconsciously extended to half an hour, under the excitement of friendly greetings and neighborly gossip. There can be no doubt that thousands every year *catch their death of cold*, to use a homely but expressive phrase, in the manner above named. Young women, especially, cannot act thus with impunity. Men perish by multitudes every year by exposures of a similar character; walking or working until they become warm, then sitting in a hall or entry or a cold counting-room; or standing still at the wharf or at a street corner; or running to reach a ferry-boat until they begin to perspire, and then sitting still in the wind while the boat is crossing. It is by inattention to what may be considered *such trifling little things* that thousands of valuable lives are sacrificed every year.

### A YOUNG GENTLEMAN,

(950) from Washington City, complained of

Uneasiness at throat, caused by repeated colds; late hours, hot rooms;
Cough most of mornings—dry, tickling, hollow;
Expectoration a little yellow;
Bloody, streaked expectoration, six months ago;
Breathing oppressed, if sit or stoop long;
Take cold easy, in every way;
Throat has various feelings, tickling, heavy aching, raw, dry, from palate to depression;
Swallowing a little difficult at times;
Voice not much affected;
Headache, costive bowels, piles occasionally;
Pain about shoulder-blades and at their points;
Soreness under both ribs sometimes;
Pains in the breast—more of a soreness from the top of the breast-bone to the pit of the stomach;
Have been ailing fifteen months;
Father, mother, sister, uncle, aunt died of Consumption.

### OPINION.

You cannot have Consumption now: you are decidedly threatened with it. With proper attention, persevering and prompt, you may ward it off effectually, and live to the ordinary term of human life to those of your occupation. It is my opinion, that without this care, you will fall into settled disease within a year.

In two months, this gentleman called to see me for the first time. His lungs were working freely and fully, over the natural standard; pulse seventy-two; appetite good; bowels regular. I did not think he required any particular medical advice; and it is my present belief, that with proper attention to diet, exercise, and regular habits of life, his health will become permanently good.

### 952.

Took a severe cold last winter, which left a severe cough. Every morning the breast feels sore, until stirs about some. Pain in the left side, running through to the left shoulder blade, and between the shoulders; pain in the breast-bone, and in the centre of the left breast. Chief complaint is pain in the chest, left side, and a constant raising of frothy, thick, tough, and yellow matter, with frequent hawking, hemming, and clearing of the throat. Age 22.

### OPINION.

Your ailments are all removeable by diligent attention to the directions I may give you. I very much hope you will spare no pains in carrying them out most thoroughly. You certainly have not Consumptive disease.

He called upon me some months afterwards, when I saw him for the first time. He had nothing to complain of; pulse sixty; his lungs working freely and fully, being considerably above the natural standard; and as far as I know, he continues well to this day.

### 973.

"Am officer in a bank. Was at a fire during Christmas, seven months ago. Used my voice a great deal; began to be hoarse; very much so by morning. This lasted a week, and went off; but in three weeks there appeared to be something about the palate which wanted to come away. Throat seemed inflamed, and ever since then have had a clogging feeling in the throat, that does not affect my voice, unless I read aloud, when I soon become hoarse. Two days ago, spit up a spoonful of dark blood; never before or since. I have a binding sensation across the top of the breast, and three months since had a pain up and down the breast-bone. Have used iodide of potash; have had the throat pencilled, and then sponged with nitrate of silver, without benefit—pulse, one hundred and ten."

### OPINION.

Yours is a throat ailment, at the entrance of the windpipe—not as low down as the voice organs. There is very considerable active inflammation there. Your lungs are a little weakened, nothing more; the pains in the breast are not serious at all, and I see no obstacle to your entire recovery.

I received letter after letter from this young gentleman, stating that no perceptible benefit seemed to follow what I advised. He was encouraged to persevere, and finally his symptoms began to change, and then disappeared; and in two months from his first consultation he wrote me to say that he had steadily improved; pulse, permanently at sixty-five; expressing his obligations, &c. This case shows strikingly the advantage of perseverance.

### A CLERGYMAN

(844) wrote to me for advice in reference to a throat complaint. I prescribed, and had entirely forgotten the circumstance, when the following letter was received:—

"I began to follow your directions on the 4th day of May, not quite three months ago, and have adhered to them strictly ever since. I am evidently a great deal better. I have lost no flesh; although it is summer, my weight has not varied three pounds since I wrote to you; it is now one hundred and forty-nine pounds. My tonsils are diminished, and give me no uneasiness, except in damp weather. From my throat, which is now generally perfectly comfortable, I am continually bringing up a pearly substance. Sometimes it is perfectly clear, and like the pure white of an egg. But this is a mighty change. At first, I could not talk five minutes in the family circle. My throat was constantly tickling and burning; so that a mustard plaster, which took all the skin off my neck in front, was a comfort; but now I can talk as much as I wish, read a page or so aloud, and am almost tempted to sing a little."

### HOW DO PERSONS GET BRONCHITIS?

In the same manner as a common cold, for Bronchitis is a common cold protracted, settling not on the lungs, but on the branches of the windpipe, clogging them up with a secretion thicker than is natural; this adheres to the inside of the tube-like branches, and to a certain extent closes them: hence, but a small portion of air gets into the lungs. Nature soon begins to feel the deficiency, and instinctively makes extra effortsto obtain the necessary quantity, in causing the patient to draw in air forcibly instead of doing it naturally and without an effort. This forcible inspiration of external air drives before it the accumulating phlegm, and wedges it more compactly in a *constantly-diminishing tube*, until the passage is entirely plugged up. The patient makes greater efforts to draw in the air, but these plugs of mucus arrest it, and there is a feeling as if the air did not get down to its proper place, or as if it were stopped short, causing a painful stricture, or cord-like sensation, or as some express it, *a stoppage of breath.* If relief is not given in such cases, either by medicine judiciously administered, or by a convulsive nature of effort at a cough, which is a sudden and forcible expulsion of such air as happened to be *on the other side* of the plug, the patient would die; and they often do feel as if they could not possibly live an hour. This is more particularly a description of an attack of Acute Bronchitis. Chronic Bronchitis is but a milder form of the same thing, very closely allied in the sensations produced, if not indeed in the very nature of the thing, to what may be considered a kind of

### PERPETUAL ASTHMA, which may in most cases be removed and warded off for an indefinite time by the use of very little medicine, if the patient could be induced to have a reasonable degree of self-denial and careful perseverance.

### HOW DO PERSONS GET CONSUMPTION ?

As they do most other diseases, by inattention, neglect, imposition on nature. Many persons have this disease hereditarily, but the same means which permanently arrest the progress of accidental Consumption will as often and as uniformly ward off, indefinitely, the effects and symptoms of the hereditary form, the essential nature of accidental and hereditary Consumption being the same. The treatment is also the same, except that in the accidental form it must be more prompt, more energetic; in the hereditary form it must be more mild, more persevering. I consider the latter, the less speedily and critically dangerous of the two.

### MISCELLANEOUS ITEMS.

A number of pages will be devoted to the illustration of a variety of topics connected with the general subject; all, however, will be of a practical character —at least, such is the intention.

CONSUMPTION IS THE OXIDATION OF THE EXUDATION CORPUSCLE. This corpuscle—*this little body*, this tubercle, this seed of Consumption—is an albuminous exudation, as minutely described on page 5, First Part, and being deficient in fatty matter, its elementary molecules cannot constitute nuclei, capable of cell development; therefore, these nuclei remain abortive, are foreign bodies in the lungs, and like all other foreign bodies there, cause irritation, tickling. This tickling is a cause of cough, as itching is a cause of scratching, both being instinctive efforts of nature to remove the cause of the difficulty. The oxidation—that is, the burning, the softening of this corpuscle or tubercle—gives yellow matter as a product, just as the burning—that is, the oxidation of wood—gives ashes as a product. Thus the yellow matter expectorated in Consumption is a sign infallible, that a destructive, consuming process is going on in the lungs, just as the sight of ashes is an infallible sign that wood or some other solid substance has been burned—that is, destroyed.

But why is it that this albuminous exudation, this tubercle, this exudation corpuscle, should lack this fatty matter, this oil, this carbon, which, did it have, would make it a healthy product, instead of being a foreign body and a seed of death ?

Consumption is an error of nutrition. The patient has soliloquized a thousand times, "I sleep pretty well, bowels regular, and I relish my food, but somehow or other it does not seem to do me the good it used to. I do not get strong." The reason of this is, that the food is imperfectly digested, and when that is the case, acidity is the result, which is the distinguishing feature of Consumptive disease. This excess of acid in the alimentary canal dissolves the albumen of the food, and carries it off into the blood in its dissolved state, making the whole mass of blood imperfect, impure, thick, sluggish, damming up in the lungs—that is, congesting them—instead of flowing out to the surface, and keeping the skin of a soft feel and a healthful warmth. Thus it is that the skin of all Consumptives has either a dry, hot feel, or a cold, clammy, dampness; at one time having cold chills creeping over them, causing them to shiver in the sun or hover over the fire; at another time, by the reaction, burning hot, the cheek a glowing red, the mouth parched with thirst. Another effect of the excess of acidity dissolving the albumen and carrying it into the blood is, that the blood is deficient in the fat, or oil, or carbon, which would have been made by the union of this albumen with alkaline secretions; the blood then wanting the fat or fuel which is necessary to keep the body warm, that which was already in the body, in the shape of what we call flesh, is used instead, and the man wastes away, just as when steamboat men, when out of wood, split up the doors, partitions, and other parts of the boat, to keep her going, she moves by consuming herself. So the Consumptive lives on, is kept warm by the burning up, the oxidation of his own flesh every day and every hour; this same wasting away being the invariable, the inseparable attendant of every case of true Consumption. He lives upon himself until there is no more fuel to burn, no more fat or flesh, and he dies—" nothing but skin and bone." What, then, must be done to cure a man of Consumptive disease ?

He must be made more (what is called) "*fleshy*;" that is, he must have more fuel, fat, to keep him warm.

The acidity of the alimentary canal must be removed, in order that the food may be perfectly digested, so as to make pure blood, such as will flow healthfully and actively through every part of the system, and become congested, sluggish, stagnant nowhere.

To remove this acidity, the stomach must be made strong, and healthfully active; but no more than healthfully active, so as to convert the food into a substance fit for the manufacture of pure blood.

To make the stomach thus capable of forming a good blood material from the aliment introduced into it, as a perfect mill converts the grain into good flour or meal, there is behind the mill a power to turn it, there is behind the stomach powers to be exerted. These are the glandular system, the liver being the main one of all. This must be kept in healthful, operating order; if it acts too much or too little, the food is badly manufactured, and the blood which is made out of the food, and of the food alone, is imperfect and impure.

After all this is done, there is one more operation, which is the last finishing touch by which pure life-giving blood is made; ☞ a sufficient amount of pure air must come in contact with it before blood is constituted. This contact takes place in the lungs; not such a contact as the actual commingling of wine and water, for the air and what is soon to become blood are not mixed together; they are kept separate in different vessels. The air is in the lungs; that is, in the little bladders or cells, and this fluid, which is to be converted into blood, is in the little veins or tubes, which are spread around over the sides of the air-cells, as a vine is spread over a wall; but these little vessels have sides so very thin, that the life-giving material of the air passes through into the blood, just as the warmth of the sun passes through glass; but while this life-giving quality of the air passes into the blood, making it perfect, the impure and deathly ingredients of the blood pass out of it, into the air, which has just been deprived of its life. Thus it is, that while the air we draw in at a single breath is cool and pure and full of life, that which is expired is so hurtful, so poisonous, at least so destitute of life, that were it breathed in, instantly, uncombined with other air, by a perfectly healthy person, he would instantaneously die. So that pure air in breathing is most essentially indispensable; first, to impart perfection, life to the blood; and also to withdraw from it its death. No wonder, then, that a plentiful supply of pure air is so essential to the maintenance of health, so doubly essential to the removal of disease and restoration to a natural condition. No wonder, then, that when a man's lungs are decaying, and thus depriving him of the requisite amount of air, he so certainly fades away, unless the decay is first arrested, and the lung power or capacity restored.

The great principles, then, involved in the cure of Consumptive disease, or, professionally speaking, the great indications, are—

To cause the consumption and healthful digestion of the largest amount possible of substantial, nutritious, plain food.

To cause the patient to consume more pure air.

To bring about the first condition requires the exercise of extensive medical knowledge, combined with a wide experience and close and constant observation. To regulate healthfully the digestive apparatus—that is, to keep the whole glandular system of the human body in healthfully-working order—requires remedies and treatment as varied in their combinations almost as the varied features of the human face. Scarcely any two persons in a hundred are to be treated in the same way, unless you can find them of the same size, age, sex, constitution, temperament, country, climate, occupation, habits of life, and manner of inducing the disease. Here are ten characteristics which are capable, as every arithmetician knows, of a thousand different combinations; so that any person proposing any one thing as a remedy—a cure for Consumption, applicable to all cases and stages, must be ignorant or infamous beyond expression.

The two things above named will be always curative in proportion to their timely accomplishment. The ways of bringing these about must be varied according to constitution, temperament, and condition. The *mode* of doing the thing is not the essential, but *the thing done.* Beyond all question, the thing can be done: Consumption can be cured, and is cured in various ways. The scientific practitioner varies his means according to the existing state of the case. The name of the disease is nothing to him; he attacks the symptoms as they are at the time of prescribing; and if he be an experienced practitioner, he will know what ought to be done, and how it should be attempted, just as a classical scholar knows the meaning of a classical phrase or word the first time he ever sees it as perfectly as if he had seen it a thousand times before. And without setting myself up as an instructor to my medical brethren, I may here intimate my conviction, that the cure of Consumption would be a matter of every day occurrence, if they would simply study the nature of the disease, read not a word of how it had been treated by others, but observe closely every case, and treat its symptoms by general principles, as old as the hills, and follow up the treatment perseveringly, prescribe for the symptoms, and let the name and disease go. But then they must first understand perfectly the whole pathology of the disease—its whole nature. That, however, requires years of laborious study and patient observation.

The above things being true, as perhaps none will deny, it is worse than idle to be catching up every year some new medicine for the cure of Consumption. The readiness with which every new remedy is grasped at, shows beyond all question that the predecessors have been failures. Scores of cures have been eagerly experimented upon;—naphtha, cod liver oil, phosphate of lime, each will have its day, and each its speedy night, simply because no one thing can by any possibility be generally applicable, when solely relied upon. The physician must keep his eye steadily upon the thing to be done, varying the means infinitely, according to the case in hand. Therefore, the treatment of every individual case of Consumption must be placed in the hands of a scientific and experienced physician in time, and not wait, as is usually the case, until every balsam and syrup ever heard of has been tasted, tried, and experimented upon, leaving the practitioner nothing to work upon but a rotten, ruined hulk, leaving scarcely anything to do but to write out a certificate of burial, and receive as compensation all the discredit of the death.

The intelligent reader will perceive that I have spoken of the cure of Consumption as a matter of course. From the resolute vigor with which cod liver oil has been prescribed and (believingly) swallowed within a very few years past, one would suppose that almost every one believed that the cure of Consumption was a common every day affair. A few years ago, nobody thought so, except perhaps here and there a timid believer who kept his credence to himself, lest he should be laughed at. But the public got hold of the idea that cod liver oil was a remedy for the cure of Consumption, and swallowed thousands of barrels of what was said to be it, before they thought of inquiring for the facts of the case. I have never to this hour heard or read of a single case of true Consumption ever being perfectly and permanently arrested by the alone use of cod liver oil. No case that I have seen reported as cured would bear a legal investigation. There has always been some kind of reservation. It is my belief that all the virtues of cod liver oil, or any other oil, or phosphate of lime, as curative of consumption of the lungs, are contained in plain meat and bread, pure air and pure water; the whole of the difficulty being in making the patient competent to consume and assimilate enough of these. Herein consists the skill of the practitioner, and on this point he needs to bring to bear the knowledge, the study, the investigation, the observation, the experience of a life-time; and he who trusts to anything short of this, throws his life away.

The following articles are interesting and corroborative. "Littell's Living Age," No. 379, for August, the most popular and best conducted journal of the kind in America, copies from the London "Spectator" the following highly interesting and well-written article. Every line of it merits the mature consideration of the intelligent reader.

### "NEW HOSPITAL FOR DISEASES OF THE CHEST.

"While one-third of the deaths in the metropolis are ascribable to diseases of the chest, the hospital accommodation devoted to that class of diseases has heretofore been only one-tenth; that is to say, the most prevalent and destructive class of diseases has had the least counteraction among the poorer classes. This peculiar, if not studied neglect, must be ascribed to a notion, now happily dying out, that diseases connected with the respiratory organs, and especially the lungs, were virtually beyond the reach of certain or effective treatment. It was indifference to this old notion that Lord Carlisle made an admission, in his address to Prince Albert, on laying the first stone of the City of London Hospital for Diseases of the Chest—'We admit,' he said, 'that hospitals ought to give the preference to those maladies which afford a prospect of cure, rather than to those of a less hopeful character.' Now this admission, especially as compared with the qualification which followed it, that very much may be effected by precaution and a timely counteraction, is far too strong for the truth. Without accepting as literally true the inference of a physician eminent in the treatment of pectoral diseases, that all persons are at one time or other visited by maladies of that class, we believe it is certain that the proportion of mortality, enormous as it is, scarcely represents the comparative extension of such diseases. In the practical and popular sense of the word, it may be said that cure is as common in the class of pectoral diseases as in any other class. It has become much more common, indeed, since the great advance that has been made with the knowledge of such complaints in our own day. This advance has been of a two-fold character. The immense progress of physiological inquiry has thrown great light on the connection and common causes of most cognate diseases, not only with each other but with the general health, and has thus enormously augmented the power of the physician in treating them by medicine and regimen. The invention of the stethescope, by placing the exploration of the inner chest within reach of observation, has given a distinctness of knowledge on the most characteristic and dangerous symptoms, heretofore unattainable: it has thus completed the round of evidence which establishes the connection of diseases, and at the same time guides the nature and application of topical treatment.

In discovering that the prevalency of pectoral diseases was far greater than had been supposed, science has also discovered how much more they are under subjection to the general laws of physiology and medicine. This branch of science, however, is younger than others—a fact which teaches us to remember how much is to be expected from the active and vigor ous intellects now devoted to its exploration. We may also remember that while the primary object of hospitals is the relief of sufferers who are too poor to obtain it for themselves, they are also great instruments for the benefit of society at large, by checking the inroads of disease where it could not otherwise be encountered. They are still more signally valuable as great schools for the study of the diseases to which they are appropriated. They exemplify most powerfully the double blessing of charity, for him that gives as well as him that receives; the aid extended by a hospital to the poor is returned to the rich in the knowledge which it collects; for in rescuing from untimely death the assembled children of poverty, science learns, as it could in no other way do, methods which enable it to rescue the children of wealth.

The more hopeful character of the most modern science had been in great part anticipated by the brave intellect of Andrew Combe. Before his time, it was too generally, if not universally assumed, that the symptoms of Consumption were a death-warrant; he proclaimed the reverse truth, and established it. He became in his own person the teacher and exemplar, both to physician and patient; and in his compact popular volume and regimen, he has recorded, in a form accessible to all, the conclusions of his practical experience. He did away many of the old coddling notions, which helped to kill the patient by stifling the pores of the skin, filling the lungs with bad air, softening the muscular system with inaction, and deadening the vital functions; a service scarcely more useful in reconciling the patient to the restorative influences of nature, than in returning hope to the afflicted relatives, and in showing what might be done by common sense and diligence. At an early age, Andrew Combe was found to be in a Consumption—words which were formerly accepted as a death-warrant, in submission to which the awed patient duly laid down and died; Andrew Combe lived more than twenty years longer, a life of activity, usefulness, and temperate enjoyment.

"The 'People's Journal,' for July, one of the most popular European publications, has an interesting article in relation to the Consumption Hospital, founded at Brompton; and few institutions have risen so rapidly. It has a long list of noble and wealthy subscribers, with the Queen and most of the royal family at its head. 'As death has abundantly proved the mortality of the disease, so, paradoxical as it may seem, death also supplies us with evidence that the chief structural lesions of Consumption, tubercles in the lungs, are not necessarily fatal. The writer of these lines can state, from his own observation, (which has not been limited, and is confirmed by that of others,) that, in the lungs of nearly one-half of the adult persons examined after death from other diseases, and even from accidents, a few tubercles, or some unequivocal traces of them, are to be found. In these cases, the seeds of the malady were present, but were dormant, waiting for circumstances capable of exciting them into activity, and if such circumstances could not occur, the tubercles gradually dwindled away, or were in a state of comparative, harmless quiescence. This fact, supported by others, too technical to be adduced here, goes far to prove an important proposition, that Consumptive disease is fatal by its degree, rather than by its kind; and the smaller degrees of the disease, if withdrawn from the circumstances favorable to its increase, may be retarded, arrested, or even permanently cured. There are few practitioners of experience who cannot narrate cases of supposed Consumption which, after exhibiting during months and even years, undoubted symptoms of the disease, have astonished all by their subsequent, more or less, complete recovery. Cautious medical men have concluded themselves mistaken, and that the disease was not truly tuberculous; but, in these days, when the detection and distinction of diseases is brought to a perfection bordering on certainty, the conclusion that recoveries do take place from limited degrees of tubercles of the lungs, is admitted by the best authorities, and is in exact accordance with the above-mentioned results of cadaveric inspection. Consider properly, and you will be ready to admit the truth of what has been already established by experience, that Consumption may be often prevented, arrested, or retarded by opportune aid. On this point we know that many medical men are utterly incredulous, and stigmatize others who are less so, in no measured terms; but, with the present rapid improvements in all the departments of medical knowledge, there is less ground for such incredulity than there was for that which opposed and ridiculed Jenner in his advocacy of vaccination as the preventive of small-pox.'

In view of the above and other testimonials of the most distinguished living writers in favor of the curability of Consumption, it is impossible for any well-informed and well-balanced mind any longer to deny it. We cannot conceive it possible that so many great men should be so much deceived on a point which they have made it the business of a life-time to investigate and study.

### "SUICIDE BY STARVATION.

"A very curious example of suicide by means of starvation occurred some years ago in Corsica. During the elections, the Sieur V. rushed into the electoral college armed with a dagger, which he plunged into the breast of a man who had done him some injury. The man fell dead at his feet. The assassination was committed in the full light of day, and in the presence of an assembled multitude.

"V. was tried, found guilty, and condemned to death. His high spirit and resolute character were well known, and it was suspected that he would seek, by a voluntary death, to evade the disgrace of perishing on the scaffold. He was therefore vigilantly watched, and every precaution taken to deprive him of the means of putting an end to his existence.

"He resolved to starve himself to death during the interval which elapsed between the sentence of the Court or Assizes and the reply which the Court of Cassation would make to the appeal he had addressed to it.

"He had succeeded in concealing from the observation of his jailers a portion of the food with which they supplied him, so as to make it be believed that he regularly took his meals. After three days' abstinence, the pangs of hunger became insupportable. It then suddenly occurred to him that he might the more speedily accomplish the object he had in view by eating with avidity. He thought that the state of exhaustion to which he was reduced would unfit him to bear the sudden excess, and that it would inevitably occasion the death he so ardently desired. He accordingly sat down to the food which he had laid aside, and ate voraciously, choosing in preference the heaviest things. The consequence was that he was seized with a violent fit of indigestion, from which, contrary to his expectation, the prison doctor speedily cured him.

"He then resumed his fatal design. He suffered again what he had undergone before. The torture was almost beyond his strength. His thirst, too, was intolerable. It overcame his resolution. He extended his hand towards the jug of water which had been placed in his cell. He drank with avidity, and, to use his own expression, *was restored to life.*

"To avoid yielding again to a similar temptation, he daily took the precaution of overturning the jug of water which was brought to him. Lest he should be induced to raise it to his lips, he threw it down with his foot, not venturing to touch it with his hand. In this manner he passed eighteen days.

"Every day, at different intervals, he noted down in his album a minute account of his sensations. He counted the beatings of his pulse, and marked their number from hour to hour, measuring with the most scrupulous attention the gradual wasting of his strength. In several parts of his melancholy *memento*, he declares that he felt it harder to bear the agonies of thirst than those of hunger. He confesses that he was frequently on the point of yielding to the desire of drinking. He nevertheless resisted.

"He was surprised to find his sight become more and more clear, strong, and accurate; it appeared to him like the development of a new sense. The nearer he approached his latter moments, the more his power of vision seemed to increase. On this subject he thus expresses himself: 'It appears as though I could see *through* the thickest walls.' His sense of feeling likewise attained the most exquisite sensibility. His hearing and smelling improved in a similar degree. His album contains many curious statements on these subjects.

"The Sieur V. had devoted some attention to anatomy and physiology; and he attributes the increased acuteness of his senses to the way in which the intestinal irritation acted on the nervous system.

"His ideas, he says, were numerous and clear, and very different from anything he had experienced in moments of excitement or intoxication. They were all directed to logical investigation, whether he applied them to an analysis of material objects, or to philosophic contemplation. He also felt himself inspired with a singular aptitude for mathematical calculation, a study for which he had previously felt very little inclination. In short, he declares that he never derived so much gratification from his intellectual condition, as throughout the whole duration of his physical torture.

"He made notes in his album to the last moments of his existence. He had scarcely strength sufficient to hold the pencil with which he traced the following words: 'My pulse has nearly ceased to beat—but my brain retains a degree of vigor which, in my sad condition, is the greatest solace Providence could bestow on me. It is impossible that I can live out this day. My jailers watch me, and fancy they have adopted every precaution. They little think that I have outwitted them. Death annuls the sentence which has been pronounced on me. In another hour, perhaps, they will find nothing but a cold corpse.'

"V. expired as he foretold. His album has been carefully preserved. It is a record replete with interest to medical professors. The slow torture, endured with so much courage, and described with such remarkable clearness, renders it one of the most curious documents in the annals of medical science."

Illustrating the same point, a gentleman, Mr. I. F. H., stated to the author that he was once under medical treatment for some affection of the eyes, requiring a very scanty diet. His general health was excellent, but he was always hungry; yet so far from having any sense of debility, he had, when he went out into the street, an elasticity of mind and body, an instinctive desire of locomotion, which caused him to feel as if he could almost fly, and a joyousness of spirit, which was perfectly delightful.

These two cases strikingly show, that with a smaller amount of food, and consequently of blood, men are cheerful in mind and active in body; ☞ therefore, a small amount of food, perfectly digested, gives more health and strength than a larger, not so. It is better, incomparably better, to feel a little hungry all the time, than to feel full, oppressed, heavy, with over eating.

Every patient of mine, who ever expects to get well, must keep this fact constantly and practically in view. It is too much the custom to measure one's health by the avidity of his appetite and his increase in flesh, as if he were a pig; forgetting that a voracious appetite and fat are always indications of a diseased body. A uniform moderate appetite is the attendant of good health. A racer's ribs must be seen before he is fit for the track, because then he is most capable of endurance.

The next incident shows, that with a moderate amount of substantial food and cold water, such being prisoner's fare, men may live for many years, with but little exercise, in the dark vaults of a prison, breathing all the time an atmosphere not very pure, as may be readily supposed. And it is earnestly hoped that the incidents narrated will leave upon the mind of every reader a life-long impression as to the value, both to the sick and the healthy, of living habitually on a moderate allowance of plain, substantial, nourishing food. It may be well to recollect here that it is not the quality, so much as the quantity of food, which lays the foundation every year of innumerable diseases and deaths. Let it be remembered, also, that men need a variety of food; living on one or two kinds for a length of time will always undermine a healthy constitution. Milk only has all the elements of life; and any other one kind of aliment, used indefinitely as to time, will as certainly deteriorate the constitution, bodily and mental, as anything that is planted will deteriorate if kept for successive years in the same field unrenewed. The popular notion that one or two kinds of food at a meal is *most wholesome*, is wholly untrue. On the contrary, several kinds at a meal, other things being equal, are more conducive to our well-being. Quantity, and not quality, is the measure of health.

### COUNT CONFALIONERI

wrote from the great jail of Vienna as follows:—

"I am an old man now, yet by fifteen years my soul is younger than my body: fifteen years I existed, for I did not live. It was not life in the self-same dungeon, ten feet square. During six years I had a companion; nine years I was alone. I never could rightly distinguish the face of him who shared my captivity in the eternal twilight of our cell.

"The first year we talked incessantly together. We related our past lives, our joys forever gone, over and over again.

"The next year we communicated to each other our ideas on all subjects.

"The third year we had no ideas to communicate; we were beginning to lose the power of reflection.

"The fourth, at intervals of a month or so we would open our lips, to ask each other if it were indeed possible that the world were as gay and bustling as it was when we formed a portion of mankind.

"The fifth year we were silent.

"The sixth, he was taken away, I never knew where, to execution or to liberty. But I was glad when he was gone: even solitude was better than that pale and vacant face. After that, I was alone.

"Only one event broke in upon my nine years' vacancy. One day, it must have been a year or two after my companion left me, my dungeon door was opened, and a voice, I knew not whence, uttered these words: 'By order of his Imperial Majesty, I intimate to you, that one year ago your wife died.' Then the door was shut. I heard no more. They had but flung this great agony in upon me, and left me alone with it again."—Phil. Pennsylvanian, March 2, 1850.

Having shown the bearing which food has on health, I desire to make some statements as to the value of air and exercise in the same direction. These will be given succinctly, in the hope that the intelligent reader will study them and apply them at length, especially if he should come to me for medical advice. My habit is not merely to cure when I can the patient who comes to me, but to induce him to study and understand his own case and constitution, so that by the application of general principles he may afterwards be able to regulate his health under all ordinary circumstances, as far as it can be done by diet, air, exercise, and regularity of personal habits; but never venturing to take an atom of medicine, however simple, except by the special advice of an educated, experienced physician.

### IMPORTANCE OF PURE AIR TO HEALTH.

Men are reported to have lived three weeks without food, but without air we cannot live three minutes. The lungs of a full-sized man weigh about three pounds, and will hold twelve pints of air; but nine pints are as much as can be inhaled at one full breath, there being always a residuum in the lungs; that is, *all* the air that is within them can never be expelled at once. In common, easy breathing, in repose, we inhale one pint. Singers take in from five to seven pints at a single breath. We breathe, in health, about eighteen times in a minute; that is, take in eighteen pints of air in one minute of time, or three thousand gallons in twenty-four hours.

On the other hand, the quantity of blood in a common-sized man is twenty pints. The heart beats seventy times in a minute, and at each beat throws out four tablespoons; that is, two ounces of blood: therefore, there passes through the heart, and from it through the lungs, an amount of blood every twenty-four hours equal to two thousand gallons.

The process of human life, therefore, consists in there meeting together in the lungs, every twenty-four hours, two thousand gallons of blood and three thousand gallons of air. Good health requires this absolutely, and cannot be long maintained with less than the full amount of each; for such are the proportions that nature has ordained and called for. It is easy, then, to perceive, that in proportion as a person is consuming daily less air than is natural, in such proportion is a decline of health rapid and inevitable. To know, then, how much air a man does habitually consume, is second in importance, in determining his true condition, to no other fact; is a symptom to be noticed and measured in every case of disease, most especiallly of disease of the lungs; and no man can safely say that the lungs are sound and well and working fully, until he has ascertained, by actual mathematical measurement, their capacity of action at the time of the examination. All else is indefinite, dark conjecture. And I claim for myself to have been the first physician in America who made the measured amount of consumed air an essential element as to symptoms, in ascertaining the condition of persons in reference to the existence of Consumptive disease, and making a publication thereupon. The great and most satisfactory deduction in all cases being this, that if, upon a proper examination, the lungs of any given person are working freely and fully, according to the figures of the case, one thing is incontrovertibly true, demonstrably true, that whatever thousand other things may be the matter with the man, he certainly has nothing like Consumption. And Consumption being considered a fatal disease by most persons, there is quite a willingness to have anything else; and the announcement and certainty that it is not Consumption, brings with it a satisfaction, a gladness of relief, that cannot be measured.

On the other hand, just in proportion as a person is habitually breathing less air than he ought to do, in such proportion he is falling fast and surely into a fatal disease. This *tendency* to Consumption can be usually discovered years in advance of the actual occurrence of the disease; and were it possible to induce the parents of children over fifteen years of age to have investigations as to this point in the *first place*, and *then* to take active, prompt, and persevering measures to correct the difficulty, and not one case in a thousand need fail of such correction, with but little, if any medicine, in most instances many, many a child would be prevented from falling into a premature grave, and would live to be a happiness and honor to the old age of those who bore them. Persons who live in cities and large towns think, and wisely so, that the teeth of their children should be carefully examined by a good dentist once or twice a year; but to have the condition of the lungs examined, and, if need be, rectified, who ever thought of such a thing? And yet, as to practical importance, it immeasurably exceeds that of attention to the teeth. The latter are cared for as a matter of personal appearance and comfort: the lungs are a matter of life and death. We can live and be happy without a tooth, but without lungs we must prematurely die. Were the condition of the lungs, after such an examination as I have suggested, a matter of opinion or conjecture only, I would not propose it; but it is not: it is a thing of numerical measurement, of mathematical demonstration, as to the one point, Do the lungs work freely and fully or not? If they do not, declining health is inevitable, sooner or later, unless their activity is restored, which, however, can be done in the vast majority of cases.

### YOUNG PERSONS.

While speaking of the health and habits of the young, it may be well further to state, that wrong indulgences debilitate the system; in time, the mind becomes unable to fix itself upon any subject profitably. Exhausting discharges further weaken the energies, and idiocy sometimes supervenes, in various forms and degrees of epilepsy; at other times, fatal symptoms of Throat-Ail and Bronchitis. (See Trousseau and Belloc.)

### A CASE.

"A youth, aged nineteen, indulged freely for some time, and at length began to experience pains about the throat. The voice was altered; shrill at first, then entirely lost. Swallowing liquids became impossible. He spit up large quantities of matter, and died after a year's illness. The lungs, on examination, were entirely sound, but the whole throat was ulcerated."

Throat-Ail and Consumption are diseases of debility, and it may be easily supposed that no progress can be made towards a cure while causes of debility *are in operation*. This statement is made here to save the necessity, in all cases, of more direct inquiries. If, however, there is no personal control, parents may apply for their children, and permanent relief be obtained without wounding the feelings or self-respect of the ailing party, who indeed may be blameless.

### MISCELLANEOUS CASES.

(851. Sept. 2.) Your lungs are unimpaired; they are in full working order. There is no tendency at this time to Consumptive disease. Your ailment is dyspeptic laryngitis, complicated with a slight pleuritic affection, and with proper attention you will get well. At the same time, it is important for you to know, that these throat affections are among the most incurable of all diseases when once fully established. This consideration should induce you to commence *at once* a proper course of treatment, and to *persevere in it* until you are *perfectly* restored to health.

*Note.*—His principal ailment was an uneasy feeling in the throat, a frequent clearing of it, and an almost constant pain in the left breast. He wrote me in three weeks, that my prescriptions were acting admirably, and that he was getting well.

(852. Sep. 2.) Your ailment is common tubercular disease, mainly tending to fix itself on the lungs, and next on the bowels. Decay of the lungs has not yet begun to take place; they are becoming inactive, about one-tenth of them doing you no efficient good. There is a reasonable probability that the disease may be arrested at this stage. A return to good health is by no means impossible; it is doubtful. The throat ailment is nothing more than what may arise from a dyspeptic condition of the stomach, liable to end in tubercular ulceration in your case, your lungs being already tuberculated to some extent; the right side slightly more than the other.

*Note.*—He complained chiefly of spitting blood, cough and debility; had been using cod liver oil for several months to no purpose. I have not heard from him since giving the opinion.

(853. Sept. 2.) You have chronic laryngitis, torpid liver, lungs acting imperfectly. There is no decaying process, no Consumptive disease, and I see no special reason why you may not, with judicious treatment, recover your health.

He complained chiefly of husky voice (had to abandon preaching), constipation, and variable appetite. In five months he wrote me that he "was able to enter upon his pastoral duties," and had been discharging them three months.

(854. Sept. 12.) Your lungs are not in a safe condition; one-third of them are now useless to you. It will be necessary for you to use diligent efforts to arrest the progress of your disease, and spare no pains in doing so.

*Note.*—Complains chiefly of spitting blood, cough, sore throat, debility. He appears to be getting well rapidly.

(855. Sept. 7.) Your disease is common consumption of the lungs; one-fourth of them are doing you no good; a part of them are irrecoverably gone; therefore, under no circumstances can you be as stout and strong as you once were. The decay of your lungs is progressing every hour. If that decay is not arrested, you cannot live until spring. Whether that decay can be arrested I cannot tell. It is possible that it may be done. It is not my opinion that it can be done.

*Note.*—Chief symptoms harassing cough, drenching night-sweats, daily expectoration of blood, constipation, irregular appetite, great emaciation and debility, could scarcely walk around one square. In three weeks he could walk twenty squares in a day without special fatigue. Here he ceased very unexpectedly to call upon me. Being a favorite child of his father, I took great interest in his case. Whether he suddenly relapsed and died, or thought he could get along now without farther aid from a physician, I do not know.

### A MERCHANT.

"At this time the lungs are untouched by disease; they do not work as free and full as they ought to do, but it is impossible that there should be any decay, or that they should be tuberculated to any extent. If your present weak state of health continues, the system will become so debilitated by winter, and so susceptible to impressions from cold, that you will in all probability fall into an eventual decline. At this time, nothing is the matter with you but symptoms arising from a torpid liver and impaired digestion. Your health can be certainly restored."

*Note.*—Aged thirty; he had spitting of blood, pains in the breast, and other symptoms which greatly alarmed himself and friends, as pointing to settled Consumption. He got perfectly well with little or no medicine, and remains so to this day.

On the same day, September 18, a young woman came for examination, having walked several squares.

*Opinion.*—"You are in the last stages of Consumption. A large portion of the lungs is utterly gone; the decay is rapidly progressing, and nothing can arrest it. Death is inevitable before the close of the year."

*Note.*—She had a hoarse, loud cough, cold feet, chills, no appetite, irregular bowels, difficult breathing on slight exercise. I did not prescribe. She died in a short time.

(714.) J. S., married, aged 40, an officer in the Mexican war, and severely wounded at Cerro Gordo, complained most of *cough, weakness, sweating at night*, and *shortness of breath*. Any sudden movement of the body or mental emotion produced almost entire prostration. Had lost one-ninth of his weight.

*Opinion.*—"Your lungs are in good working order; no decay, not an atom; the yellow matter expectorated is a morbid secretion from the windpipe and its branches. Your heart is affected; the calibre of its blood vessels is too small to transmit the blood with sufficient rapidity; hence the fluttering and great debility on any sudden motion or protracted exercise, for these but increase the quantity of blood to be conveyed away. Your ailments depend on constitutional causes to a great extent, and in proportion are capable of removal."

I heard of this gentleman no more for one year, when he came into my office a well man in every respect, saying that he began to get well in three days after taking the first weekly pill, and thought as he was doing so well, there was no necessity of writing.

A case (988) similar, in some respects, is now under treatment: great throbbing of heart and weakness on slight exercise; a violent beating in the temples the moment he lays his head on a pillow at night. This does not occur when he lies on his back. Frequent numbness and pricking sensation in left arm and leg; tosses and tumbles in bed for hours every night before he can get to sleep; great general weakness, and total inability to walk; riding in any kind of a carriage over a rough road, often but not always, brings on sick headache; has frequent distress at stomach; pulse one hundred; much dispirited, and has fallen away more than one-sixth.

*Opinion.*—"Your ailment is a symptomatic heart affection, depending now, mainly, on constitutional causes, originating in over efforts of mind and body. The lungs are sound and well."

In three weeks he writes, each of the two weekly pills brought away large quantities of stuff, yellow as yolk of egg, with masses of a colorless, stringy substance, and left my bowels regular. I now sleep as well as I could wish; very little pain in the side; stomach no longer distresses me. I have gained strength, but no flesh, and some throbbing yet remains.

*Note.*—This man will probably get well if he continues to follow the directions as well as at the beginning. He had been advised to exercise his arms and the muscles of his chest a great deal, and was told that he must work, and thinking he could accomplish both at the same time, and being naturally industrious, he began to saw wood for family use during the coming winter; but every day he became weaker and worse, until he could scarcely stand up. This being a heart affection, every moment of such exercise necessarily aggravated the malady.

This shows the mischievous effects of taking a wrong view of a case and of following the advice of every person one meets with. Many persons are advised to death. Over-confident advice is the attendant of inexperience and ignorance. It is forgotten that unpaid advisers, being well themselves, do not endanger their own lives, in case their recommendations are inefficient, if, indeed, not positively hurtful. Many are infatuated with *vegetable remedies*, taking it for granted that they can do no harm, even if they do no good; forgetting that in many cases a loss of time is equivalent to a loss of life, and that the most virulent poisons in all nature—those which produce almost instantaneous death—are of vegetable origin, such as nicotine, prussic acid, and the like.

I. Q. H., married, aged forty-eight; had a distressing cough, which, with a severe pain below the point of the right shoulder-blade, prevented any refreshing sleep. He arose every morning sweaty, haggard, and weary; no appetite, and daily expectoration of large quantities of matter. He had fallen off forty-two pounds, and was greatly depressed. I informed him that his lungs were not diseased, and that there was no necessary obstacle to his recovery. His friends thought he became worse under my treatment, for at the end of four weeks he was confined to his bed day and night, with frequent rigors and flushes. The pain steadily increased, at times aggravated almost beyond endurance by a cough, which I thought nothing could safely control, and hence gave nothing for it. He thought he could not live unless speedily relieved; his relative, a physician, came to remonstrate against my "holding out hopes of recovery to a man who was evidently sinking with Consumption." I informed the patient he was better; that he would probably need no more medicine, and explained to him the reasons for such an opinion. In a few days his strength began to increase, and he walked out. He left the city soon afterwards, and now, at the end of three years, he is a hearty, healthy man, weighing upwards of two hundred pounds, having taken no medicine since he saw me. I considered his case to be one of great torpidity of the liver, with abscess, and treated it accordingly. The reader may see by this, how important it is sometimes to know that a case is not Consumption, and also the value of a steady resistance against ignorant interferences.

(July 23.) "Your lungs are not diseased, nor are they even impaired in their action. There is not only no Consumption in your case, but there is a less tendency that way than in most persons. You have not merely lungs enough for the ordinary wants of the system, but a large amount in reserve. Your whole aliment is a dyspeptic condition, and there is no reason why a rational habit of life should not restore you to as good health as you have ever enjoyed, without any medicine whatever."

He complained of pain in the breast, large expectoration, voice sometimes husky, and a tightness across the chest.

(July 23.) "Your lungs at this time are not in a satisfactory condition, more than one-sixth of them being valueless to you. A portion at the top of the right breast has decayed away. Your case is one presenting all the ordinary symptoms of common Consumption. It will be altogether impossible for you to arrest the progress of your disease if you continue your present habits of business (printer). If you pursue an out-door calling, and acquire judicious habits of life, it is probable that your disease may be arrested, and that you may be restored to renewed health."

*Note.*—As he had a good appetite, was working daily at his trade, and did not feel very bad, he thought it not advisable to abandon his calling, and died in three months.

(Nov. 8.) "Your lungs are whole, sound, and in full working order. There is at present no appearance of Consumptive disease. Your ailments arise wholly from general constitutional causes, and may be removed by proper and rational habits of life and conduct."

*Note.*—He was not satisfied with my opinion; was fully impressed with a belief that he was falling into a decline, and insisted upon repeated examination. He was a man of wealth, of fortunate social relations, and very naturally dreaded death—too much so for a man. He observed faithfully the directions given, no medicine was advised, and wrote in three months that he was as well as he ever was in his life; his chief complaint was an "uneasy sensation about the heart," and some "trouble in the throat."

(Nov. 9.) "Your lungs are not diseased materially at this time. They do not work fully, but there is no decay. Your ailment is Chronic Laryngitis, of a very dangerous and aggravated character. It is very doubtful whether you will get well. Something may be done for you by a rigid attention to all the directions given."

*Note.*—He could not speak above a whisper; swallowed food with great difficulty and pain. He remained under the treatment of his family physician, and died in seven weeks."

(849.) "You are suffering under the combined influence of dyspepsia and consumptive disease, and they mutually aggravate each other. One-fifth of your lungs are now useless to you. This is a very serious deficiency. The extent to which you may be benefited, can only be ascertained by attention to directions given. Your case is not hopeless, yet it is critical and of a very grave character." He died in five weeks. He could not or would not control his appetite, and the author ceased to prescribe, as is his practice when instructions are not implicitly followed.

(Aug. 30.) "All your ailments arise from a want of natural proportion between exercise and eating. If these were properly regulated, you would get well without any other means, as the lungs are sound, healthy, and entire. You are too full of blood, and it is not healthful; hence it does not flow freely, but gathers about the internal organs, oppressing them and giving rise to any number of ailments, constantly varying as to character and locality. Make less blood, and take more exercise, according to the printed instructions given you, and your return to good health will be speedy and permanent."

She complained of pains and oppressions, particularly about the chest, tickling cough, &c. I heard no more of her for six months, when her husband, a Southern planter, called to express his satisfaction, and to say that she was in good health, and had been for some time.

(Sep. 30.) "Your disease is common consumption of the lungs. It began at the top of the right breast, and after making some ravages there, it ceased and attacked the left, which is now in a state of continued decay. It may spontaneously cease on the left side, as it did on the right; in that event, life would be preserved for the present. Without such an occurrence as just named, one-half of the lungs being useless to you, the constitution usually fails in six or eight weeks, and sometimes much sooner." She died in six weeks.

Frail and feeble persons often outlive by half a life-time the robust and the strong, because they feel compelled to take care of themselves, that is, to observe the causes of all their ill-feelings, and habitually and strenuously avoid them. Our climate is changeable, and in proportion unhealthful. In New York City, for example, during one week in December last, in which the thermometer ranged from five degrees above Zero to fifty-five, there were forty-one deaths from inflammation of the lungs, while the ordinary number is about fifteen. The healthy disregard these changes to a great extent, and perish within a few days. The feeble are more sensitive to these changes; they increase their clothing and their bedding with the cold, and with equal care diminish both, with the amount eaten, as the weather grows warmer, and thus long outlive their hardier neighbors. These precautions, with others, must all observe, THROUGH LIFE, who have been cured of an affection of the throat or lungs. Let this never be forgotten, for the oftener you are re-attacked, the less recuperative energy is there in the system, and the less efficient will be the remedial means which once cured you, unless by months of continued attention and wise observances you give the parts a power and a strength they never had before. This can be done in many cases.

But once cured, avoid the causes which first injured you. If you put your hand in the fire, you may restore it, but however magical may be the remedy, that hand will be burned as often as it is placed in the fire, without any disparagement of the virtues of the restorative. No cure of your throat or lungs will render you invulnerable. What caused the disease in the first instance will continue to cause it as long as you are exposed to them. No promise is given you of permanence of cure longer than you are careful of your health. The safer plan by far will be to consider yourself peculiarly liable to the disease which once annoyed you, and make proportionate endeavors to guard yourself habitually against its advances. All assurances that any mode of cure will afford you a guarantee against subsequent attacks, are deceptive. No medicine that any man can take in health will protect him from disease. There is no greater falsity than this, that if you are well, a particular remedy, or drink, or medicine, will fortify the system against any specified disease, whether cholera, yellow fever, or any other malady. So far from this being so, it is precisely the reverse. Doubly so; you are thrown off your guard, and in addition you make the body more liable to the prevalent malady by poisoning the blood; for whatever is not wholesome food, is a poison to the system, pure water excepted. Nothing, therefore, will protect a healthy man from disease but a rational attention to diet, exercise, cleanliness, and a quiet mind; all else will but the more predispose him to it. But when once diseased and then cured, these things are not sufficient to keep him well; he must avoid what first made him an invalid, otherwise permanent health is not possible, but a speedy relapse and death are inevitable, as to Throat-Ail, Bronchitis, and Consumption.

### DANGER OF CUTTING TONSILS.

M. Landouville removed an enlarged tonsil of a woman, aged 21. In eight days she had uncontrollable spitting of blood, which was constant, besides vomiting a large quantity. Small pulse; extremities cold. The danger was imminent. Various means had already been adopted in vain; such as ice externally, styptics internally; then pressure with lint dipped in lemon juice; but it was at length controlled by pressing ice against the spot with forceps. (See *Hays' Med. Jour.*, October, 1851.) Other cases are given in medical publications; they are not of frequent occurrence, but each one operated upon is liable to experience disagreeable results. An operation is seldom necessary—not one case in twenty. And as in the case above, the danger was not over for a week after the operation had been performed, others who have the tonsils taken out have cause for a lengthened and most unpleasant suspense.

It must not be forgotten that Throat-Ail is in very many instances wholly unmanageable, and ends fatally, simply from its being thought lightly of, until it has produced such a state of general irritation throughout the system, that the constitutional stamina is exhausted, and the pulse is habitually a fourth, or third, or even more, above the natural standard. Most generally, such cases go on to a fatal termination, in spite of all modes of treatment. This is so uniformly the result, that any certain benefit in such cases cannot be promised; nor is it just that the general principles of treatment should suffer discredit from failure here; they are admirably and uniformly successful whenever they are applied in the early stages of the disease. It is to invoke prompt attention to the first and earliest symptoms of Throat-Ail, that pains have been taken in these pages to describe them plainly, clearly, and distinctly.

### CELL DEVELOPMENT.

The human body is in constant transition. The particles of which its structure is constituted are not the same in position and relation for any two minutes in succession. Thousands of atoms which compose it the present instant are separated from it the next, to make a part of it no more; and other thousands, which are a portion of the reader's living self while scanning this line, will have been rendered useless and dead on reading the next. There are two different armies of workers, whose occupations cease not from the cradle to the grave. One army, composed of its countless millions, is building up the body; the other removes its waste; one party brings in the wood and the coal for the fire-place and the grate, the other carries away the ashes and the cinders;—the builders and the cleansers. When the builders work faster than the cleansers, a man becomes fat, and over-fat is a disease. When the cleansers are too active, the man becomes lean, and wastes away to a skeleton, as in Consumption. Health consists in the proper equilibrium of these workers.

Every movement of the body, every thought of the mind, is at the expense of a portion of the material frame; that is to say, certain atoms of the living body are killed by every action of the mind, by every motion of the body, and being dead, are useless. But they must be removed from the body, or these "heaps of slain" would fill up the workshop of life, and the whole machinery would stand still; the fire-place would be filled with ashes, the furnace clogged with cinders, and the grate be useless. Vast masses of these dead atoms are pushed, worked out, or thrown from the body at the surface. At any night, on undressing, the cleanliest person may rub from the body countless numbers of these dead atoms, a teaspoon-ful of them may be gathered from the feet at a single washing, if long neglected. Hence the value of thorough daily frictions to the skin, as promotive of health, because, on an average, we all eat about one-third more than is needed; thus throwing on the cleansers a third more labor every twenty-four hours than they were designed to perform. By the frictions we come to their aid artificially. They are wise who perform these frictions daily and well; but wiser they by far who do not eat the extra one-third, and consequently do not need to be scrubbed and bathed and washed every day of their existence, to save them from the effects of over-feeding. Better eat less and save trouble. The surplus third would feed half the poor of the land.

But a larger portion of these dead atoms are scattered in the more interior parts of the body, and the cleansers remove them by first rendering them fluid, as solid ice or snow is made fluid by heat. It is then, as it were, sucked up by these cleansers, and conveyed finally to the blood, just at the heart, where they are mingled together and sent direct to the lungs, where they meet with the pure air that is breathed. Here an exchange takes place between the air and the blood. The air gives to the blood its oxygen, its life, while the blood gives its death to the air. Hence it is that the air gives life as it goes into the lungs, but gives death if breathed unmixed as it comes from the lungs; that is, if a healthy person were to breathe for three minutes no other air than that which has just come out o[illegible] the lungs of another man, in three minutes [illegible] would die. Hence my insisting so much on causi[illegible]

Consumptive persons to breathe the largest possible amount of pure air; it unloads the blood more perfectly of its dead atoms, and also gives life to the essence of food which it also meets in the lungs; that is, puts the finishing work to its becoming living blood.

Let us notice next the builders, whose work is to supply new and living particles as fast as the old ones fall off and die. These new particles are in the blood, which delivers its living freight as it flows through the body, as a steamer delivers its freight to the thousand different ports as it ploughs along the majestic Mississippi. Whenever a living particle comes to the point where it is needed to supply the place of one just fallen or dead, by some inscrutable, inexplicable agency, as quick as electricity itself, a vesicle, a cell, a little boat, as it were, is formed, which floats it to the spot, delivers its charge, and bursts and dies, its duty done, the object of its creation having been performed:—an apt type of the whole and living man, who, when the great object of his creation is performed on earth, himself passes away in death; and happy indeed would he be, were that work so fully, so well, and so invariably done. These little wrecked, these bursted boats, have been collected, and ascertained to be made invariably and almost wholly of two materials—phosphorus and lime, which also are constituents of the brain itself. This phosphorus and lime are supplied by what we eat and drink. If we do not eat and drink enough, or if what we do eat and drink has not enough of these constituents; or if, again, it is not perfectly digested, then there is not enough of these constituents to make the necessary boats to freight the nutrient particles to their destination; hence, the man wastes away to skin and bone, and dies—not because he does not eat, but because what he does eat does him little or no good. Especially thus is it in Consumption; a man dies of inanition, or, as physicians say, *an error of nutrition.*

Consumptive people die for want of strength, want of flesh, want of nutriment; not for want of lung substance, as is almost universally supposed. They die, in almost every instance, long before the lungs are consumed, so far as to be incapable of sustaining life. Numerous cases are given where men have lived for years with an amount of available lungs not equal to one-fourth of the whole. They were there, perhaps, but not available, not efficient. The majority of persons who die of Consumption, perish before a *third* of the lungs have consumed away, in consequence of loose bowels, torpid liver, indigestion, night sweats, want of sleep, clogging up of the lungs with matter and mucus by the daily use of cough drops, balsams, tonics, or other destructive agents. These symptoms need but be controlled to protect life indefinitely; that is to say, if the symptoms were prescribed for according to general principles, and properly nursed, letting the Consumptive portion of the disease alone, it would sometimes cure itself, or at least allow the patient to live in reasonable comfort for a number of years.

The reader may almost imagine that he has a clue to the cure of Consumption, if he could but give the patient phosphorus and lime, or phosphate of lime—that is, burnt bones—eight or ten grains, with the first mouthful of each meal, so as to let it be mixed with the food and carried with it into the blood; from twenty to thirty grains being daily needed in health. The scientific world were charmed less than a hundred years ago by the discovery of oxygen. It was supposed that as oxygen was the constituent of the air which imparted vitality to the blood, gave it its purity, its activity, and filled the man with life and animation, nothing was needed but to take enough oxygen to purify the blood, and thus strike at the root of all disease. Accordingly, the oxygen was prepared and administered. The recipient revived, was transported, was fleet as the antelope, could run with the wind. He smiled, he fairly yelled for joy, and—died, laughing, or from over excitement. The machine worked too fast; it could not be stopped, and pure oxygen has never been taken for health since.

Thus it will, perhaps, always be with artificial remedies; they cannot equal those which are prepared in Nature's manufactory. The phosphate of lime, in order to answer the purposes of nature, must be eliminated from the healthful digestion of substantial food in the stomach, and the only natural and efficient means of obtaining the requisite amount is, to regulate the great glands of the system in such a manner as to cause the perfect digestion of a sufficient amount of suitable food, ☞ and this is within the power of the scientific practitioner, in the great majority of cases of Consumption, when attempted in its early stages; but for confirmed Consumption—that is, when the lungs have begun to decay away, it is criminal to hold out any promises of cure, or even of essential relief, in any given instance.

It is often stated as disparaging to physicians, that, notwithstanding the general increase in knowledge, in all departments, and the claim that medicine is reduced almost to a science, that human life is gradually shortening. There is great reason why men should not live so long as formerly. As a nation, we live more luxuriously; our habits of eating and sleeping have become more artificial, more irregular. Large numbers of people have no regular occupation. Our young women are trained in female boarding schools, which, with rare exceptions, are academies of mental, moral, and physical depravation; where novel reading in secret, and a smattering of everything in public, with a thorough practical knowledge of nothing, is the order of the day. From graduation to marriage nothing is done to establish the constitution, to make firm the health—no instructions given as to how that health may be preserved, no active teaching as to household duties, no invigorating morning walks, no wholesome, elegant, and graceful exercises on horseback. The days are spent in eating, in easy lounging, in ceremonial visitings, in luxurious dreaminess over sentimental fictions; their nights in heated rooms or crowded assemblies of hot and poisoned, if not putrid air. No wonder that with educations like these, the girls of our cities and larger towns fade away into the grave long before they reach the maturity of womanhood.

Our young men, also, in cities and large towns especially, grow up in too many instances without any stamina of constitution. Bad practices—drinking, chewing, smoking, theatre going, secret society gatherings—involving late hours, late suppers, late exposures, private indulgences—these destroy the health, deprave the morals, and waste the energies of the whole man. Many are permitted to grow up without any trade, trusting to a wealthy parentage, or political influence, or the name of a profession, entered only for show and not for practical life. Others grow up as clerks in stores, banks, offices, with good salaries it may be; but when the merchant has become a bankrupt, the offices failed, the banks broken, the party in power defeated, their occupation is gone, their resources are exhausted; they lounge about waiting for a place, the clothes are wearing out, the board bill is in arrears, independence lost, spirits broken, mind irritated, disposition soured, and the first crime is committed—that of engaging board without any certain means of paying, or leaving a struggling widow in arrears;—the proud, the high-minded, the well-dressed, courteous, and cheerful-faced young man of six months ago has made his first step towards degradation, by making a toiling woman give him for nothing the bread and meat which she had earned in toil and sweat, and tears perhaps, and which the children of her own bosom needed. When the honor is lost, low habits and loss of health and life soon follow. Let every young man from the country hesitate to come to the city to try his fortune, unless he have *learned well* an honest and substantial trade; then he may work his way sternly and steadily to usefulness, influence, and wealth. It is for want of a suitable education and occupation that such numbers of our young go down to a premature, if not dishonored, grave. But notwithstanding these errors as to the education and employment of our young men and young women, medical writers have been extensively disseminating useful knowledge by means of books, pamphlets, lectures, newspaper articles and the like, in reference to the preservation of health in the nursery, the school-house, the academy, the college—in factories, workhouses, penitentiaries, as to diet, exercise, ventilation, drains, sewerages, house-building; and the general result is, that within three hundred years past, the average length of human life has been increasing and not diminishing. The average age increased two and a half years for the twenty years ending 1820 in the United States. For the fifty years ending in 1831 in France, it increased from 28¾ years to 31½, notwithstanding the devastations of the wars of Napoleon and the French Revolution. In London, for the century ending 1828, the average age of all who died had increased 4¾ years. In Geneva, 300 years ago, it was 21 years; it is now 41.

Europe is computed to have a population of two hundred and thirty millions. Not a hundred years ago, Gibbon, the great historian, estimated it at less than one-half. This immense increase has taken place notwithstanding the millions who have emigrated to this and other countries—notwithstanding, too, the far greater drawback, that during a considerable portion of the time the most desolating wars were waged that were ever carried on there. This can only be accounted for by the reforms which medical science has introduced, and the more general diffusion of practical knowledge as to the preservation and promotion of health, in publications made by eminent physicians and surgeons.

As, therefore, a higher degree of medical intelligence has extended the average of human life—in some places fifty per cent., taking all diseases together—it is reasonable to suppose that increased intelligence as to one class of diseases would, in the course of time, have a like happy effect; that if more truthful views as to the nature, *causes*, and symptoms of diseases of the lungs were extensively promulgated among the people, their fearful ravages would be diminished in corresponding proportion.

In 1851, the deaths in Boston, from Consumption alone, were about thirty per cent. of the entire mortality, and the Medical Association announces that it "is steadily on the increase from year to year." If this is the case in Boston, where such large quantities of cod-liver oil have been purely made, and hence more easily and cheaply obtained, it presents a striking and practical contradiction of its curative powers in Consumption, and calls upon us in louder and louder tones to look less to the cure of this terrible scourge, and more to the detection of its early symptoms and its prevention, by scattering intelligence to every family, and on the wings of every wind, as to what are its causes and what these early symptoms are. Such is the object of this publication.

*Patent Medicines* are those whose contents are not made known. A physician who has any respect for himself would scarcely use them, or advise their use. It is a universal custom among all honorable practitioners, to communicate to their brethren any valuable discovery: thus, any one of them is benefited by the discoveries of all the others: they hold their knowledge in common. A remedy discovered to be truly valuable in New York to-day, in the cure of any disease whatever, is, in a few months, known wherever the English language is read and spoken. Thus thousands, scattered over the world, whom the discoverer never could see, are benefited and blessed by his discovery, through the regular practitioner. Some other person obtains this knowledge, prepares the ingredients, disguises them with some inert substance, and sells it as a secret remedy, leaving those to die, as far as he cares, who do not buy from him or his agents; while thousands of others, in other states and countries, perish for the want of a knowledge locked up in his bosom. Any patent medicine is a cure for a given disease, or it is not. If it is not a cure, it is false and criminal to sell it as a cure. If, on the other hand, it is what it professes to be, it cannot be much better than murder to withhold it from those who cannot purchase it, and to allow thousands, at a distance, to die from the want of it, who never heard of it, or, if they did, live too far away to send for it in time. Let those who purchase these articles think of the argument, and aid and abet no more, by their patronage, those who allow their fellow-creatures to die by thousands every year, who would be saved (if what is said be true) by the knowledge of the remedy whose composition is so carefully concealed.

Many things have been passed over in the foregoing pages, which might satisfy the curiosity or interest a large class of readers, but it is not necessary that they should be known, and if known, might have an injurious effect, considering the present state of knowledge on the subject of Consumptive disease; such, for example, as stating what symptoms are infallibly fatal, what kind of persons, as to sex, temperament, color of hair, eyes, skin, make of body, are most liable to it, or having it, have less hope of recovery. For similar reasons, I have given but few fatal cases and their symptoms; for persons having one or more of these same symptoms might conclude that they, too, must die, when those same symptoms, in combination with others, would indicate a very different result. I do not wish the reader to suppose that I do not lose any cases—that few or none die in my hands. I lose patients as other physicians do. I have lost some whom I expected would recover. Nor do I wish to make the impression, that it is a frequent occurrence that persons in the advanced stages of Consumption are restored to comparative health; for it is not a frequent occurrence—it is a rare thing. My object is, first, to show what the early symptoms are; and, second, to induce the reader to make application to me at this early stage, with the full assurance of my belief, that thus one person would not die of disease of the throat or lungs where one hundred now do. In truth, I had greatly rather that persons in the advanced stages would not apply to me; for it at once involves a degree of responsibility and solicitude, which is to extend through weeks and months, and for which any money paid is not the shadow of a remuneration.

I greatly desire it to be understood that I have no magical means of cure. Ailments of the throat and lungs are not to be removed by a box of pills or a bottle of balsam. It is not the work of a day, nor of a week. These cases often require weeks and months of treatment, and of a treatment constantly varying, to meet the varying phases of the disease. Sometimes it occurs, but not often, that a person writes for advice in full, and it is given, and the single prescription, PERSEVERED IN, has effected a happy cure, and months and years after, such persons have come to see me, to express their gratification. At other times, prescriptions are sent, and the persons never heard of afterwards. In nearly all cases, these are young people, or persons who have no energy of character, no perseverance, no determination. For a few days or a fortnight, they give a general attention to the directions, and because they are not cured, break off and apply to some other physician, to follow the same course, or become negligent of themselves, and eventually die. It is a most hopeless task to attempt to cure any of Throat-ail or Consumption who have no energy of character. It is time, and trouble, and money lost, as they are not diseases to be eradicated in a day, by a drop or a pill. It is to be accomplished, if at all, by a determined, thorough and persevering attention, for weeks and sometimes many months, to rational means, ☞ calculated to build up the constitution, with a decreasing use of medicine and an increasing attention to habits of life.

ASTHMA.—I have said but little of this distressing disease. It is not often critical or dangerous until advanced life. As a general rule, it is incurable. Children who have it, sometimes *grow out of it*. In some women, it often disappears at the turn of life; in others, during the years of child-bearing. A *fit of asthma*, as it is called, generally cures itself, by being let alone. An attack is often hastened away by judicious means. In persons of a feeble constitution, it is liable to come or go any day or hour, and prove fatal in marked changes of weather—that is, to very cold, or from cold to a warm, heavy, thawy, foggy atmosphere. The only proper and efficient method of treatment is, to *prevent the attack*, which can be done in the great majority of cases, and for an indefinite length of time. The distinguishing symptom is *want of breath*; the patient feels sometimes as if it would almost kill him to speak two or three words; the necessity of breath is so great, he cannot find time to cough, and represses it, lest it should *take his breath away*. He can neither cough, sneeze, spit, nor speak freely. He sits up, wheezes, throws his head back, wants the doors and windows opened. The attacks generally come on towards the close of the day, and pass off about midnight or soon after, when the cough becomes loose, and large quantities of a substance more or less yellow, pearly, and tenacious, are expectorated; urination becomes copious, and the patient recovers, to be attacked in the same way night after night, until the violence of the disease is expended, and recovery takes place; or if these ameliorations do not occur about midnight, the case is aggravated, and the patient dies in a few hours. This disease is treated more at length in the large edition. It is certain, that in a vast number of cases, whether hereditary or accidental, the attacks can be indefinitely warded off by proper care and habits of life, if the constitution is not much broken.

### CROUP OF CHILDREN.

Many a lovely child is destroyed in a single night by this alarming disease. Its nature is described in the First Part. It is a disease of the windpipe, which is filled or lined with a phlegm, which becomes more and more tough, almost leathery—thickens, and at length closes up the passage to the lungs, and the child dies.

It usually comes on in the night. The distinguishing symptom is a wheezing, barking cough. A mother who has ever heard it once, needs no description to enable her to recognise it again. The first born are most likely to perish with it; simply because the parent has no experience of its nature, and hence is not alarmed in time, or knows not what to do, while the physician is being sent for. In the hope of being instrumental in saving some little sufferer, whose life is inexpressibly dear, at least to one or two, I will make some suggestions, not for the cure of the patient, but to save time. The instant you perceive that the child has *Croup*, indicated by the *barking Cough*, *uneasy breathing*, *restlessness*, send for a physician, and as instantly wrap a hot flannel around each foot, to keep it warm; but while the flannels are being heated, dip another flannel, of two or more thicknesses, in spirits of *turpentine*, or spirits of *hartshorn;* or have a large mustard plaster applied, one that will reach from the top of the throat down to some two inches below the collar bones, wide enough at top to reach half-way round the neck on either side, and nearly across the whole breast at bottom. But it will take time to send for a physician, to prepare flannels, and to make the plaster or obtain the turpentined flannel, and in some cases fifteen minutes is an age—is death, if lost; therefore, while these things are preparing, give the child, if one year old or over (and half as much, if less), about half a teaspoon-ful of Hive Syrup, and double the dose every fifteen minutes until vomiting is produced; and every half hour after vomiting, give half as much as caused the vomiting, until the physician comes, or the child ceases to cough, when he breathes free, and is safe. If you have no Hive Syrup, give a easpoon-ful of Syrup of Ipecac, and double the dose every fifteen minutes until vomiting is produced. If you have been so thoughtless as to have nothing at all, boil some water, keep it boiling, dip a woolen flannel of several folds into it, squeeze it out moderately with *our hand*, and apply it as hot as the child can possibly bear to the throat, and in from one to three minutes, according to the violence of the symptoms, have another to put on the instant the first is removed, and keep this up until the breathing is easy and the cough is loose and the phlegm is freely discharged, or until the arrival of the physician.

I wish to impress upon the reader's mind a few disconnected subjects. Consumption most generally comes on by a slight cough in the morning, about the time of rising or first stirring about. The existence of tubercles in the lungs is not necessarily fatal; they remain dormant for a life-time, unless irritation or inflammatory action is excited by *bad colds neglected*, or *exhausting habits or diseases, or debilitating occurrences, or wasting indulgences.* These things throw more persons into fatal Consumption than are destroyed by the hereditary form of the disease; and these should be, as they can in very many instances, safely remedied.

The following recipes are frequently referred to:—

*How to Toast Bread.*—Keep the bread a proper distance from the fire, so as to make it of a straw color. It is spoiled if it is black, or even brown.

*Toast Water.*—Take a slice of bread about three inches across and four long, a day or two old. When it is browned, not blackened, pour on it a quart of water which has been boiled and afterwards cooled. Cover the vessel, and after two hours, pour off the water from the bread gently. An agreeable flavor may be imparted by putting a piece of orange or lemon peel on the bread at the time the water is first poured on the bread.

*Barley Water.*—Take two tablespoons of pearl barley, wash it well in cold water, then pour on it half a pint of water, and boil it fifteen minutes; throw this water away, then pour on two quarts of boiling water, and boil down to a pint; then strain it for use. An ounce of gum arabic dissolved in a pint of barley water is a good demulcent drink.

*Flax-seed Tea.*—Take an ounce or full table-spoon of flax seed, but not bruised, to which may be added two drams of bruised liquorice root; pour on a pint of boiling water, place it covered near the fire for four hours, strain through a cotton or linen rag. Make it fresh daily.

*Tamarind Whey.*—Two tablespoon-fuls of tamarind, stirred in a pint of boiling milk; then boil for fifteen minutes, and strain.

*Wine Whey.*—Take a pint of milk, put it on the fire; as soon as it begins to boil, pour on eight or ten tablespoons of Madeira wine, in which has been stirred two teaspoons of brown sugar; stir the whole until it has been boiling for fifteen minutes; then strain through a cloth.

*Boiled Flour and Milk.*—Take a pint of flour; make it into a dough ball with water; tie it tightly in a linen bag; put it into a pan of water, covering the ball, and let it boil ten hours; place it before the fire to dry, cloth and all; take it out of the cloth, remove the skin, dry the ball itself. Grate a tablespoon of this, and stir it into a pint of boiling milk, until a kind of mush is formed.

*Boiled Turnips.*—Small turnips boiled make one of the best articles of food which invalids and convalescents can use. Carrots may be added; half and half. Boil them once; repeat the boiling in fresh water until they are quite soft; press the water out through a coarse cloth; then mix enough new milk to form a kind of pulp; season with salt, and then place them before the fire until it is a little dry or crusted.

*Beef Tea.*—Cut into thin slices a pound of lean meat, pour on a full quart of cold water, let it gradually warm over a gentle fire; let it simmer half an hour, taking off the skum; strain it through a napkin. Let it stand ten minutes, then pour off the clear tea.

*Cracked Wheat.*—Dry some common wheat, then grind it in a coffee mill; boil it three or four hours; add a little salt, a little milk, butter, cream, or molasses may be added, as in using homminy. It should be always washed clean, and then boiled long enough to become of the consistence of boiled rice or homminy. A pint of wheat dried and groound is enough for a day; not to be used for supper.

*Dandelion Diet Drink.*—Take three ounces of the bruised root of the dandelion flower, which should be gathered in July, August, and September; pour on a quart of water, boil it to a pint, and strain it.

| | | |
|---|---|---|
| 60 Drops | make | one Teaspoon. |
| 4 Teaspoons | " | one Tablespoon. |
| 2 Tablespoons | " | one Ounce. |
| 2 Ounces | " | one Wine-glass. |
| 2 Wine-glasses | " | one Gill or Teacup. |
| 4 Gills | " | one Pint. |

---

I greatly desire that nothing I have written should excite unreasonable expectations as to the speediness of cure of the diseases treated of; they come on slowly, are sometimes for years gathering force in the system, and hence it is unreasonable to suppose that they are to be eradicated except by energetic treatment, long-continued, unless attended to in their very first stages. The patient, page 107 top of second column, expressed himself as being cured in two days:—it was three months before every remnant of disease seemed to have left his throat. Remember this, if no other sentence—attend at once to the first morning cough, or frequent hawking, hemming, swallowing, or want of clearness of voice of two weeks' continuance; otherwise, in nine cases out of ten, a fatal Consumption will be the result.

W. W. HALL.

42 Irving Place, New York.

